# Supplementary material for: Pyruvate Dehydrogenase Kinase 1 inhibition mediated oxidative phosphorylation enhancement in cartilage promotes osteoarthritis progression
Source: BMC Musculoskelet Disord. 2023 Jul 20;24:597. doi: 10.1186/s12891-023-06585-6 (PMC10357736; doi:10.1186/s12891-023-06585-6)
Supplement: Supplementary file 1 — Additional file 1. [file 12891_2023_6585_MOESM1_ESM.zip › KEGG.Gsea.1653622432464/KEGG_MATURITY_ONSET_DIABETES_OF_THE_YOUNG.html]

Details for gene set KEGG\_MATURITY\_ONSET\_DIABETES\_OF\_THE\_YOUNG[GSEA]

|  || Dataset | 55457\_collapsed\_to\_symbols.cls.cls #OA\_versus\_normal.cls.cls #OA\_versus\_normal\_repos |
| Phenotype | cls.cls#OA\_versus\_normal\_repos |
| Upregulated in class | OA |
| GeneSet | KEGG\_MATURITY\_ONSET\_DIABETES\_OF\_THE\_YOUNG |
| Enrichment Score (ES) | 0.19826514 |
| Normalized Enrichment Score (NES) | 0.65463763 |
| Nominal p-value | 0.93317425 |
| FDR q-value | 0.99688995 |
| FWER p-Value | 1.0 |
Table: GSEA Results Summary

  

Fig 1: Enrichment plot: KEGG\_MATURITY\_ONSET\_DIABETES\_OF\_THE\_YOUNG      
 Profile of the Running ES Score & Positions of GeneSet Members on the Rank Ordered List

  

| SYMBOL | TITLE | RANK IN GENE LIST | RANK METRIC SCORE | RUNNING ES | CORE ENRICHMENT || 1 | HHEX | hematopoietically expressed homeobox [Source:HGNC Symbol;Acc:HGNC:4901] | 270 | 0.774 | 0.0841 | Yes |
| 2 | PAX6 | paired box 6 [Source:HGNC Symbol;Acc:HGNC:8620] | 304 | 0.746 | 0.1829 | Yes |
| 3 | NEUROD1 | neuronal differentiation 1 [Source:HGNC Symbol;Acc:HGNC:7762] | 1008 | 0.515 | 0.1983 | Yes |
| 4 | HNF4G | hepatocyte nuclear factor 4 gamma [Source:HGNC Symbol;Acc:HGNC:5026] | 2509 | 0.302 | 0.1228 | No |
| 5 | PAX4 | paired box 4 [Source:HGNC Symbol;Acc:HGNC:8618] | 3011 | 0.249 | 0.1177 | No |
| 6 | SLC2A2 | solute carrier family 2 member 2 [Source:HGNC Symbol;Acc:HGNC:11006] | 3168 | 0.234 | 0.1373 | No |
| 7 | ONECUT1 | one cut homeobox 1 [Source:HGNC Symbol;Acc:HGNC:8138] | 3443 | 0.207 | 0.1442 | No |
| 8 | INS | insulin [Source:HGNC Symbol;Acc:HGNC:6081] | 3885 | 0.166 | 0.1325 | No |
| 9 | NEUROG3 | neurogenin 3 [Source:HGNC Symbol;Acc:HGNC:13806] | 4443 | 0.118 | 0.1052 | No |
| 10 | FOXA2 | forkhead box A2 [Source:HGNC Symbol;Acc:HGNC:5022] | 5021 | 0.071 | 0.0701 | No |
| 11 | HNF4A | hepatocyte nuclear factor 4 alpha [Source:HGNC Symbol;Acc:HGNC:5024] | 7160 | -0.093 | -0.0833 | No |
| 12 | MNX1 | motor neuron and pancreas homeobox 1 [Source:HGNC Symbol;Acc:HGNC:4979] | 7956 | -0.152 | -0.1245 | No |
| 13 | IAPP | islet amyloid polypeptide [Source:HGNC Symbol;Acc:HGNC:5329] | 7976 | -0.153 | -0.1051 | No |
| 14 | NKX2-2 | NK2 homeobox 2 [Source:HGNC Symbol;Acc:HGNC:7835] | 8977 | -0.230 | -0.1515 | No |
| 15 | HNF1B | HNF1 homeobox B [Source:HGNC Symbol;Acc:HGNC:11630] | 9072 | -0.237 | -0.1266 | No |
| 16 | HNF1A | HNF1 homeobox A [Source:HGNC Symbol;Acc:HGNC:11621] | 9621 | -0.285 | -0.1305 | No |
| 17 | PKLR | pyruvate kinase L/R [Source:HGNC Symbol;Acc:HGNC:9020] | 10120 | -0.333 | -0.1239 | No |
| 18 | GCK | glucokinase [Source:HGNC Symbol;Acc:HGNC:4195] | 10137 | -0.335 | -0.0797 | No |
| 19 | PDX1 | pancreatic and duodenal homeobox 1 [Source:HGNC Symbol;Acc:HGNC:6107] | 10409 | -0.362 | -0.0516 | No |
| 20 | NKX6-1 | NK6 homeobox 1 [Source:HGNC Symbol;Acc:HGNC:7839] | 11314 | -0.473 | -0.0576 | No |
| 21 | NR5A2 | nuclear receptor subfamily 5 group A member 2 [Source:HGNC Symbol;Acc:HGNC:7984] | 11362 | -0.481 | 0.0041 | No |
| 22 | HES1 | hes family bHLH transcription factor 1 [Source:HGNC Symbol;Acc:HGNC:5192] | 12676 | -0.849 | 0.0174 | No |
Table: GSEA details [plain text format]

  

Fig 2: KEGG\_MATURITY\_ONSET\_DIABETES\_OF\_THE\_YOUNG      
 Blue-Pink O' Gram in the Space of the Analyzed GeneSet

  

Fig 3: KEGG\_MATURITY\_ONSET\_DIABETES\_OF\_THE\_YOUNG: Random ES distribution      
 Gene set null distribution of ES for **KEGG\_MATURITY\_ONSET\_DIABETES\_OF\_THE\_YOUNG**
